# Enhancing Lower Third Molar Surgery: Using The Piezoelectric Technique for Superior Postoperative Outcomes and Complication Prevention

**DOI:** 10.3390/dj12110353

**Published:** 2024-11-01

**Authors:** Stefania Cantore, Fábio França Vieira e Silva, Maria Eleonora Bizzoca, Annafrancesca Smimmo, Lorenzo Lo Muzio, Gisela Cristina Vianna Camolesi, Mario Pérez-Sayáns, Andrea Ballini

**Affiliations:** 1 Department of Precision Medicine, University of Campania Luigi Vanvitelli, 80138 Naples, Italy; stefania.cantore@unicampania.it (S.C.); fabio.francavieiraesilva@unicampania.it (F.F.V.e.S.); annafrancesca.smimmo@unicampania.it (A.S.); 2 Oral Medicine, Oral Surgery and Implantology Unit (MedOralRes), Faculty of Medicine and Dentistry, University of Santiago de Compostela, San Francisco Street, s/n, 15782 Santiago de Compostela, Spain; giselacristina.vianna@rai.usc.es; 3 ORALRES Group, Health Research Institute of Santiago de Compostela (FIDIS), Santiago de Compostela University Clinical Hospital, University of Santiago de Compostela, Choupana Street, s/n, 15706 Santiago de Compostela, Spain; mario.perez@usc.es; 4 Department of Clinical and Experimental Medicine, University of Foggia, Via Rovelli, 50, 71122 Foggia, Italy; lorenzo.lomuzio@unifg.it (L.L.M.); andrea.ballini@unifg.it (A.B.); 5 Medical Statistics Unit, University of Campania “Luigi Vanvitelli”, 80138 Naples, Italy

**Keywords:** third molar surgery, piezoelectric technique, post-surgery pain, post-surgery swelling

## Abstract

**Background:** The surgical removal of impacted mandibular third molars is routine in oral and maxillofacial surgery and common postoperative complications are widely reported in the literature. Therefore, this prospective split-mouth study aims to compare the postoperative sequelae of piezoelectric surgery versus conventional surgery of the lower third molar, focusing on pain and swelling. **Methodology:** In total, 41 patients were treated under local anesthesia and surgical removal on one side of their mouth was performed using conventional rotary surgery (micromotor) while the other side’s was by piezosurgery, with an interval of 15 days from the previous procedure (82 extraction sites); in addition, pain and swelling analyses were conducted. **Results:** The pain analysis demonstrated a median of one day of pain in patients treated with piezoelectric surgery compared to two days with conventional surgery (*p* < 0.001). The probability of not feeling pain was greater in sites treated with experimental surgery on the first and second days (*p* < 0.001). The swelling was worse in places treated with the conventional method, with the most significant difference being the distance between the angle of the mandible and the upper lip vermilion on both days and the more subtle difference between the angle of the mandible and the anterior nasal spine on the seventh day. **Conclusions:** When a piezosurgical unit is used, according to the literature, is well known that it takes more time to perform the surgical extraction of third molars. Despite that, our results show that it causes less pain postoperatively, with faster improvement and a quicker reduction in swelling compared to conventional surgery.

## 1. Introduction

Mandibular third molars, commonly known as wisdom teeth, are present in approximately 90% of the population, with 33% experiencing at least one impacted molar [1]. Both genetic and environmental factors influence the high prevalence of impacted third molars [2]. The surgical removal of impacted mandibular third molars is routine in oral and maxillofacial surgery [3]. Common postoperative complications associated with impacted third molar extraction include pain, swelling, trismus, prolonged bleeding, dry socket, alveolar osteitis, surgical site infections, abscesses, and sensory disturbances involving the inferior alveolar or lingual nerves [4,5]. The incidence of these complications varies in the literature, from 0% to 30% [4,5,6]. Numerous factors have been identified as potential risk factors for postoperative morbidity, including smoking, female gender, oral contraceptive use, and patient age [7,8].

Additionally, the position, depth, and angulation of a lower third molar can determine the difficulty of its extraction. Consequently, the level of tooth impaction represents another potential risk factor specific to the surgical procedure [9]. The Winter and the Pell and Gregory classifications, widely utilized to evaluate tooth position, provide information on a tooth’s angulation and position in the mandibular ramus, respectively, with the mesioangular position being associated with a higher risk of postoperative complications [5,10,11]. Several protocols have been evaluated to reduce the risk of complications, including different flap designs, medications, and especially instruments for osteotomy [11,12]. Traditionally, rotary cutting instruments (micromotors) are generally used for osteotomy and odontectomy due to their moderate efficiency and minimal invasiveness compared to traditional tools such as bone hammers and osteotomes. However, rotating drills can generate excessive heat, potentially leading to marginal osteonecrosis and impaired healing [13], and possible slippage can cause serious trauma to the soft tissues [14].

The philosophy of the development ultrasound methods in oral surgery using piezoelectric techniques is based on two fundamental concepts in bone surgery: the minimum invasiveness and the predictability of the surgery. The ease of control for a device can reduce bleeding during the surgery, and accurate cutting and excellent tissue healing are promising results from this type of surgery, even in some cases with anatomical complexity [15].

Piezoelectric surgery leverages the piezoelectric effect, a phenomenon where certain crystals and ceramics deform when an electric current is applied, generating ultrasonic vibrations [14,15]. This technology is especially beneficial in surgical applications because it allows for the precise and selective cutting of mineralized tissues, such as bone, while preserving the surrounding soft tissues, including nerves, blood vessels, and mucosa. The specificity of the piezosurgery technique minimizes the risk of damage to these delicate structures, thereby reducing postoperative complications [16,17]. Piezosurgery generates very small oscillations in the amplitude of 60–200 μm horizontally and 20–60 μm vertically, which is very small when compared to oscillating micro saws; thus, it provides precise and safe osteotomy cuts [16]. Furthermore, ultrasonic vibrations generated by the piezoelectric device create a microstreaming effect, which helps efficiently remove surgical debris from the site. This is further enhanced by the cavitation phenomenon, in which the rapid formation and collapse of microbubbles in the liquid produce a hemostatic effect. Also, the piezoelectric technique improves the surgeon’s visibility within the operative field [18]. The combination of these effects results in a cleaner, more controlled surgical environment, which is particularly beneficial in complex procedures like the extraction of impacted lower third molars, with safety, precision, and overall surgical outcome improvements [19,20,21,22,23].

The application of piezoelectric surgery to enhance the safety and precision of impacted lower third molar extractions was initially reported in 2008 [23].

**Figure 1:**
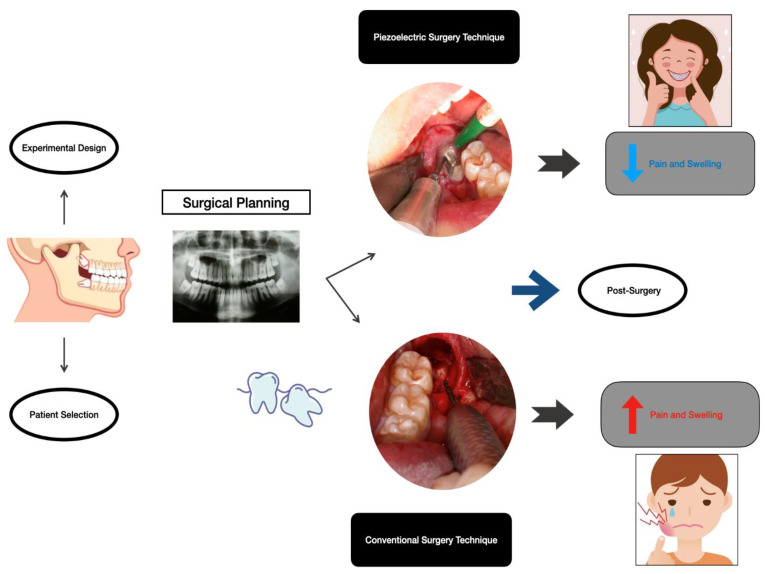
Schematic representation of the effects of the piezosurgery technique vs. conventional surgery technique in inferior impacted third molar extractions. According to the literature, piezosurgery is associated with less postoperative discomfort and leads to safer results in terms of swelling.

Since then, numerous clinical studies have assessed the effectiveness of piezoelectric surgery compared to rotary instruments for this specific procedure [20]. The current literature and some studies have greatly debated the efficiency of piezosurgery over a conventional micromotor in reducing postoperative parameters such as pain, swelling, and trismus and the intraoperative time needed for bone osteotomy. Also, biomolecular studies have shown that piezosurgery is more effective than conventional rotary instruments in reducing postoperative inflammation and oxidative stress following osteotomy [21]. Consequently, piezosurgery contributes to lower postoperative morbidity, faster recovery times, and less disruption to the patient’s quality of life (Figure 1) [22].

Therefore, this prospective split-mouth study aims to compare the postoperative sequelae of piezoelectric surgery versus conventional surgery of the lower third molar, focusing on pain and swelling over the first seven days following the procedures. Additionally, the secondary outcome is the progression of swelling in terms of the temporal distance from the surgical intervention.

## 2. Materials and Methods

### 2.1. Study Design

#### 2.1.1. Description

A prospective split-mouth study was conducted on patients who underwent bilateral lower third molar extraction surgery between April 2021 and April 2022. Each patient was treated under local anesthesia, with conventional surgery performed on one side and piezoelectric surgery on the other, 15 days after the first procedure. Moreover, we collected information on the pain at each site for each patient as a secondary outcome. The primary outcome of this study was the reduction in swelling for the piezoelectric surgery vs. the conventional one.

#### 2.1.2. Ethical Approval

The study protocol was reviewed and approved by the Comité de Ética de la Invetigación de Santiago-Lugo in Spain (Código de Registro: 2021/277). All procedures performed, as they were studies involving human participants, were in accordance with the Declaration of Helsinki.

#### 2.1.3. Inclusion and Exclusion Criteria

Inclusion Criteria: no significant systemic diseases; no clinical symptoms related to the third molar eruption (dysodontiasis) within 60 days of the surgery; no use of corticosteroids (local or systemic), non-steroidal anti-inflammatory drugs (NSAIDs), or antibiotics within 60 days of the surgery; alcohol consumption of less than 3 glasses per day; no known allergies to penicillins or non-steroidal anti-inflammatory drugs (NSAIDs); an age between 10 and 65 years; provision of informed consent for bilateral surgical intervention; similar maturation and inclusion conditions for both the mandibular third molars (teeth 38 and 48); and a healthy general condition (ASA class I or II) with complete permanent dentition. Exclusion Criteria: teeth affected by acute infections, such as pericoronitis, acute alveolar abscess, or oral submucosal fibrosis at surgery times and patients not meeting the inclusion criteria.

#### 2.1.4. Patient Selection

Patients aligned with the study inclusion criteria were defined based on their general demographic data, anamnestic elements, and radiographic and clinical characteristics. In total, 41 patients underwent bilateral lower third molar extraction, resulting in 82 extraction sites. The sample size was determined through a careful calculation using the G*Power software (version 3.1.9.7. Samsøvej, Hinnerup, Denmark). Drawing on prior research in this field, a power analysis indicated that a sample of 41 patients would provide sufficient statistical power to detect differences at a significance level.

#### 2.1.5. Patient Randomization

Each extraction site was randomly assigned to either conventional or piezoelectric surgical therapy using a simple randomization process to eliminate bias in the outcome evaluation. Patients were not informed of the technique used on each side to prevent this from influencing their perceptions of the results. To account for individual response variability, each patient received both treatments. Evaluation criteria were standardized through a patient-completed questionnaire and objective assessments were conducted pre-operatively and on the third day and seventh-day post-surgery by an operator who was unaware of the treatment assignments. The surgical team adhered strictly to the randomization protocol, with a documented allocation sequence ensuring that each technique was applied to the designated side, preserving the study’s scientific integrity and attributing the observed differences solely to the surgical techniques.

Sample stratification was based on the maturation and degree of dental inclusion of the third molars. The categories included immature dental elements fully embedded in bone (germ/bud type), mature dental elements partially embedded in bone and soft tissue (Pell and Gregory Class II B), and mature dental elements fully embedded in bone (Pell and Gregory Class III C). Bilateral extractions were performed in all patients, who were selected because their bilaterally and symmetrically impacted lower third molars required prophylactic extraction.

Pell and Gregory Class II describes teeth with half of the crown covered by the ramus, while Class III indicates teeth with the entire crown covered. Class B refers to teeth where the occlusal plane aligns with the midpoint between the occlusal plane and the cementoenamel junction of the adjacent second molar, and Class C describes teeth where the occlusal plane is below the cementoenamel junction of the second molar. The study protocol remained unchanged throughout the research. The blinding of personnel and participants was not applicable due to the nature of the intervention. Therefore, blinding was applied only to the outcome assessor.

### 2.2. Surgery Protocols

#### 2.2.1. Pre-Operative

A procedure schedule was created, allowing for a clearer assessment of pain from each procedure without the confounding effect of shared pain medication. In a split-mouth randomized controlled trial (RCT), performing surgery on both sides of the mouth on the same day presents challenges in pain assessment, particularly when different procedures are used.

#### 2.2.2. Operative and Post-Surgical Protocol

A pre-operative rinsing protocol with 0.20% chlorhexidine for one minute was adopted. The surgery was performed with the aid of truncal anesthesia without vasoconstrictor and with locoregional anesthesia of the buccinator and retromolar triangle with vasoconstrictor. A mucoperiosteal flap was carved, with the primary incision extended distobuccally to the retromolar triangle along the external oblique line, with a secondary mesial intrasulcular incision extended to the mesiobuccal gingival margin of the second molar, and a relief incision extended approximately 10 mm in the apical direction.

For the traditional technique, osteotomies and odontotomies were performed with a surgical handpiece and round (no. 6 and 8) or slotted burs, used with external cooling and operated at a low number of rotations. In cases treated with the piezoelectric technique, osteotomies and odontotomies were performed with the Easy Surgery ^®^ Bio SAF (BioSAF IN s.r.l, Trezzano Rosa (MI), Italy) instrument in Surgery mode using its dedicated tips and with abundant irrigation using saline solution refrigerated at 4 °C.

**Figure 2:**
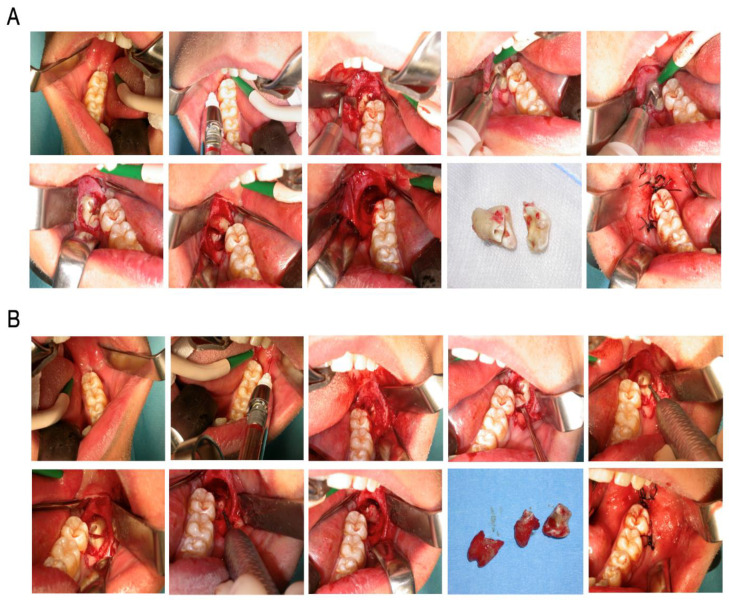
The clinical picture of the surgical procedure of bone harvesting from a retromolar area at the baseline situation and incisions similar to wisdom tooth extraction. A representative step-by-step guide for (**A**) the piezoelectric technique and (**B**) the conventional technique. Both representative pictures begin with the upper images followed by the lower ones.

The suturing was performed with 3.0 gauge BioChir ^®^ Bio SAF Biosilk threads (AgoBS 818 for the primary incision and BS 528 for the drain). The patient received a written home behavioral protocol that included external cryotherapy in the immediate postoperative period and for the following 18–24 h on day 1 (day of surgery): Rinse daily with 0.12% chlorhexidine-based mouthwash after gently brushing the area from the second to the seventh day. Abstain from any physical activity. Post-surgical systemic antibiotic therapy based on Amoxicillin + Ac was prescribed. Clavulanic acid was administered orally, with a dosage of 1 g every 12 h, and NSAID therapy based on ibuprofen granules by OS, with a dosage of 600 mg every 12 h during the first 5 postoperative days. The patient was called on days three and seven, and on this last day the sutures were removed (Figure 2).

### 2.3. Quantitative and Qualitative Variables Considered

#### 2.3.1. Pain

To facilitate patient self-assessment, this changing statistic was divided into a dichotomous variable, where 0 represented no pain or moderate pain and 1 represented severe pain. Patients with no pain or patients that held pain off using the prescribed anti-inflammatory therapy answered 0, while patients that were in pain even if they were on anti-inflammatory therapy answered 1. This categorization was chosen to mark the difference in postoperative pain, since the same antibiotics and anti-inflammatories were prescribed after both surgeries.

On the day of the operation, the patient was given a postoperative pain self-assessment scheme with a name, and space for their pain status day after day during the first 7 days, considering both sides, right and left, which would be returned, completed, in a closed envelope (so that the evaluator could not be influenced) at the end of the procedure.

#### 2.3.2. Swelling

Cutaneous region landmark measurements were taken, using a 0.0. suture thread stretched between two mosquitoes, of the distance between two easily identifiable points on both sides of the face, which underwent surgery with different techniques, and expressed in millimeters. The craniometric distances evaluated were modeled on the delta of the distance of the Angle of Jaw (AJ) and Menton (ME) (ΔAJME), Angle of Jaw (AJ) and anterior nasal spine (ANS) (ΔAJANS), Angle of Jaw (AJ) and Lower Labial Vermilion (LLV) (ΔAJLLV), Angle of Jaw (AJ) and superior labial vermilion (SLV) (ΔAJSLV), Angle of Jaw (AJ) and Nasal Wing (NW) (ΔAJNW), and Angle of Jaw (AJ) and Trago (T) (ΔAJT). The distances were recorded by the evaluating dentist before the intervention (Pre), on the 3rd day post-intervention (48 h later) (3rd day), and on the suture removal day (7th day).

### 2.4. Statistical Analysis

Descriptive variables of the population were summarized as absolute percentage frequencies for qualitative data and median and interquartile ranges (IQRs) for quantitative data. The Wilcoxon Signed-Rank Test for paired data was used to assess the difference in days of pain at sites that underwent with different treatments. Furthermore, the time to being pain-free was analyzed with life tables for each treatment. Hazard function differences were tested by the Chi-squared test.

A linear mixed-effects model was fitted to compare the postoperative progress of the experimental and conventional techniques in terms of swelling, considering the model of the delta of distances. As the random effect we chose individuals, whilst days were included as a fixed effect. This model was created using all swelling measurement differences (experimental minus conventional). A visual inspection of the residual plot did not reveal deviation from normality. All analyses were performed using R (R Core Team, 2023, Vienna, Austria) [23,24,25].

## 3. Results

### 3.1. Sample Description

Patients aged between 10 and 61 years old were included in this study, a group comprising 24 females (58.5%) and 17 males (41.5%), with a median age of 26.0 years (IQR: 18.0, 38.0). In the group with germ/bud-type dental elements, there were 28 extraction sites (34.1%) among 14 individuals (10 females, 71.4%, and 4 males, 28.6%) aged between 10 and 20 years old, who underwent simultaneous bilateral germectomy (early calcification) of their lower third molars for orthodontic or preventive reasons.

**Figure 3:**
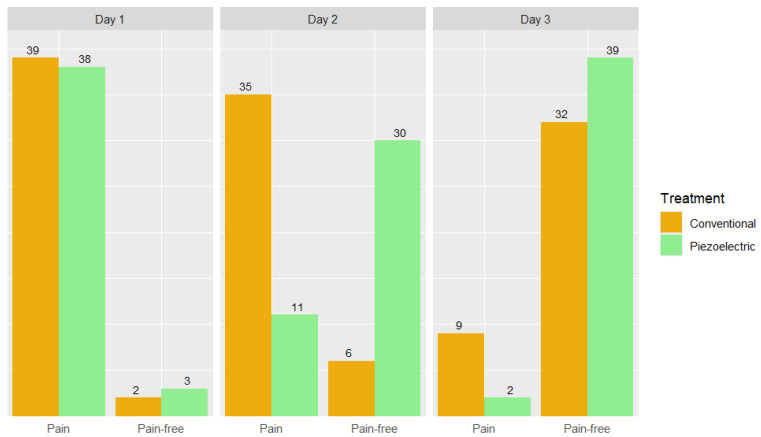
Barplot presenting pain distribution of each treatment on days 1, 2, and 3.

In the Class II B group, there were 28 extraction sites (34.1%) among 14 individuals (8 females, 57.1%, and 6 males, 42.9%) aged between 22 and 56 years old, who underwent the simultaneous bilateral removal of their lower third molars for orthodontic, prosthetic, or preventive reasons. In the Class III C group, there were 26 extraction sites (31.7%) among 13 individuals (6 females, 46.2%, and 7 males, 53.8%) aged between 21 and 61 years, who underwent the simultaneous bilateral removal of their lower third molars for orthodontic, prosthetic, or preventive reasons (Table 1 and Figure 3).

### 3.2. Pain Analysis

Firstly, the study population was stratified based on the type of surgery received to evaluate differences in postoperative pain. Notably, from day four onwards, all sites were pain-free. In Figure 2, the distribution of pain, considered as a dichotomous variable (pain/pain-free), between the two treatments was analyzed. Sites treated with the piezoelectric technique exhibited a median of one day of pain, compared to two days for sites that underwent conventional surgery. The Wilcoxon paired test confirmed a significant difference between the surgical methods (*p* < 0.001).

**Figure 4:**
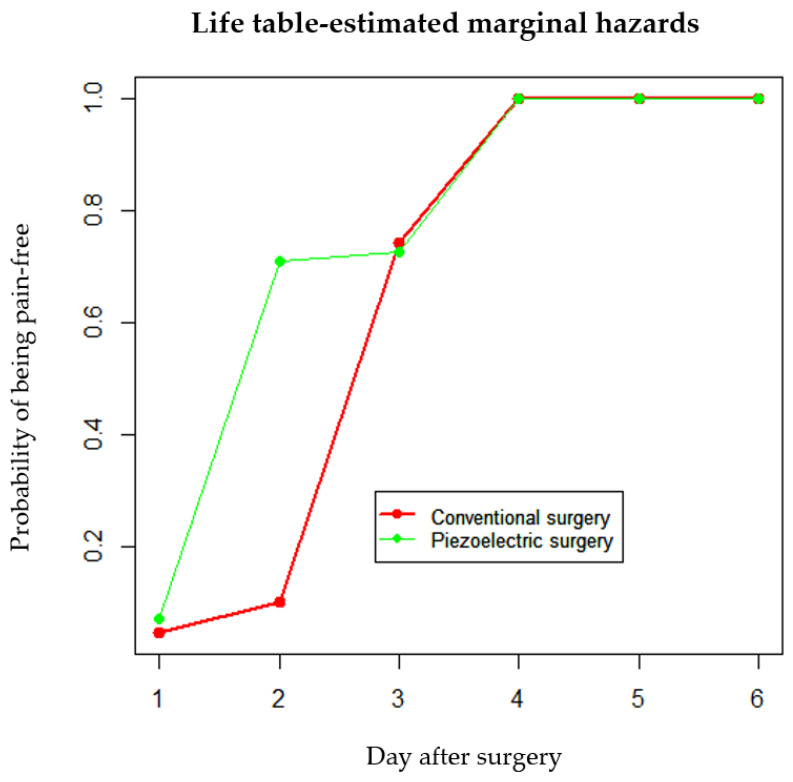
Graphical representation of the probability of being pain-free, split into conventional versus piezoelectric surgery. Estimates for each day, as marginal hazards, were calculated from the described pain tables. Note that on day 3, the estimates are influenced by the lower number of patients reporting pain.

Lastly, when considering pain as a time-to-event outcome (days to being pain-free), the hazard function for pain relief was estimated each day using the life tables method, considering the last day of pain for each patient’s treated sites. It was observed that the probability of being pain-free (that is, the marginal hazard of not having pain) was higher in sites treated with experimental surgery on days one and two. However, the estimates were similar on day three, with the conventional treatment showing a higher probability. This discrepancy is due to the lower number of piezoelectric sites still experiencing pain (the number at risk). Consequently, the difference between these two lines was statistically significant (Chi-square *p*-value < 0.001) (Figure 4).

### 3.3. Swelling Analysis

The model analyzing the changes in distance (delta) indicated that on days three and seven, the measurements were higher in sites treated with the conventional method. All deltas were negative, and the most significant difference was observed in the distance between the jaw angle and the superior labial vermilion on both days. A smaller difference was detected between the jaw angle and the anterior nasal spine on day seven (Table 2).

## 4. Discussion

Postoperative pain, facial swelling, and trismus are stressful conditions that are faced after impacted mandibular third molar surgery [18,22]. Thus, oral surgeons try to decrease postoperative complications via different approaches, such as antibacterial mouthwashes, prophylactic antibiotics, new flap designs, anti-anxiety medication, the use of corticosteroids and/or anti-inflammatory drugs, and the use of piezosurgery [1,2]. Piezoelectric surgery has been extensively documented in the literature, highlighting its significant benefits in third molar surgeries [22]. Among the primary advantages is its ability to perform the precise and selective cutting of mineralized tissues and preserve the integrity of soft tissues, including nerves, blood vessels, and mucous membranes. Furthermore, numerous studies evaluating the efficacy of this surgical technique report notable improvements in postoperative outcomes such as pain, trismus, and swelling [14,15,16,17,18,19].

Compared with traditional surgery using rotary bur techniques, piezosurgery is initially more time-consuming due to the slower micrometric cutting action of the piezoelectric device. However, as surgeons gain more experience with ultrasonic osteotomy, surgery times tend to decrease. Therefore, although the piezoelectric technique is associated with longer surgery times at first, we believe that with increased experience and continuous improvements in the technique, the duration of piezosurgery procedures will be significantly reduced [20,21].

Although the topic has been covered extensively in the literature, Cicciù et al. [22], in their meta-analysis, highlighted the high heterogeneity in published studies, and their trial sequential analysis showed a failure to reach the size threshold of the information required for a Z curve, suggesting that more high-powered studies are needed to draw definitive conclusions. Hence, the present study was undertaken to assess and compare the surgical and postsurgical outcomes of third molar removal using piezoelectric surgery and a rotary bur.

In our study, the pain analysis conducted regarding the comparison between piezoelectric surgery and conventional surgery demonstrated a median of one day of pain with the experimental technique, compared to two days for sites undergoing conventional surgery (*p* < 0.001). Furthermore, the probability of not feeling pain was greater in sites treated with experimental surgery on the first and second days (*p* < 0.001). However, the estimates were similar on the third day, with conventional treatment presenting a greater probability of no pain. However, this discrepancy is explained by the low number of patients who underwent piezoelectric surgery who still had pain on the third day. From the fourth day onwards, all patients no longer reported pain from either technique.

In agreement with our results, Mantovani et al. [26] also observed that the average assessment of pain reported by patients was significantly lower in patients undergoing dental extraction by piezosurgery; equally, in all cases, the reported postoperative pain assessed was higher on the day of surgery and progressively improved day after day until day 6 after surgery. Similar results were found in studies by Barone et al. and Sivolella et al. [27,28]; however, there was no statistical significance in this study relating to the pain analysis.

In contrast, Rullo et al. [17] observed that there were only better results in pain analyses when an odontectomy was performed with a piezosurgical instrument for cases with a simpler extraction; in cases with a more complex extraction, postoperative pain was significantly greater in the piezosurgical group.

In our study, the analysis of swelling using a model that analyzes changes in distance (delta) showed that both on the third and seventh day, the measurements were greater in the places treated with the conventional method, with the most significant difference being in the distance between the angle of the mandible and the upper lip vermilion on both days and a more subtle difference being seen between the angle of the mandible and the anterior nasal spine on the seventh day.

Piersanti et al. [29] demonstrated through their study that the postoperative surge calculated before surgery and 7 days postoperatively using digital calipers was significantly lower when using piezosurgery compared to the conventional technique. Similar results were described by Mantovani et al. [26]., who demonstrated a clinical value of facial swelling on day 7 of 1.10 in the rotary group versus 1.02 in the ultrasound group (*p* < 0.005).

Although many benefits of piezoelectric surgery have already been described, one of the major drawbacks reported in the literature is the increased operation time required for bone preparation. In a randomized prospective crossover clinical study, Sivolella et al. [28] demonstrated that a lower third molar piezoelectric osteotomy took 9.4 min longer than one with rotary instruments. Despite this, patients report that the piezoelectric technique improves comfort and generates low vibration and noise, which can minimize psychological stress and anxiety [26,29].

Another disadvantage of the ultrasonic system is the significant learning curve required [30]; despite its selective cutting capability, applying a higher pressure at the tip can impede the cutting efficiency of the insert and release the energy as heat, potentially damaging the bone and adjacent soft tissue [31,32].

On the other hand, from a biological point of view, Ueki et al. [33] evaluated the inferior alveolar nerve through neurosensors (sensitivity recovery) after a bilateral sagittal split osteotomy using piezosurgery. They observed the anatomical integrity of the inferior alveolar nerve in all cases. The use of an ultrasonic device in bilateral sagittal split osteotomy facilitated the neurosensory recovery of the inferior alveolar nerve. The results indicated a fast return of sensitivity and preservation of the anatomical integrity of the inferior alveolar nerve with the application of piezoelectricity.

Chiriac et al. [34] explored the impact of piezoelectric osteotomy on intraoral bone morphology, cell viability, and differentiation. Their study found that autogenous bone particles collected with ultrasound contained vital cells capable of differentiating into osteoblasts, unlike those collected through conventional osteotomies.

Postoperative complications following third molar surgery can significantly impact a patient’s quality of life (QoL) [35]. Various methods have been employed to assess this, but obtaining relevant results remains challenging [36,37]. Metenziletoglu et al. [38] found no significant difference in QoL between their piezosurgery and rotary instrument groups during their first postoperative week. Both groups had high QoL scores (77.33 ± 14.49 for the piezosurgery group and 77.17 ± 13.63 for the rotary instrument group), indicating that both techniques produced acceptable outcomes.

Cicciù et al. [22] recently highlighted significant biases in published studies on this subject and emphasized the need for new research to address existing gaps in the literature. In this context, our study provides consistent results regarding the comparative analysis of pain and swelling between conventional and piezoelectric techniques for lower third molar extraction. It is important to acknowledge the lack of an assessment of healing as a limitation of our study. However, evaluating soft tissue healing after third molar extraction is often not included in clinical studies due to several challenges that complicate an accurate assessment. The healing process of soft tissues is highly variable and influenced by numerous factors, such as individual patient health, surgical technique, and postoperative care, making it difficult to establish consistent and reliable criteria for the evaluation. The variability in healing patterns among patients, combined with the subjective nature of many assessment methods, introduces significant bias. These complexities require a focus on more straightforward and quantifiable outcome measures, such as surgical success rates and complications, which offer clearer and more manageable metrics for evaluating the effectiveness of different surgical techniques [39].

Castagna et al. [40] and Rosa et al. [41], in their respective studies, investigated the clinical outcomes of adjacent first and second molar sites after lower third molar extractions using different flap designs. Both studies aimed to assess the impact of these flap designs on postoperative periodontal parameters, including the plaque index (VPI), probing pocket depth (PPD), and clinical attachment loss (CAL). Rosa et al. found no significant differences between the various flap designs at different follow-up intervals, suggesting that the choice of flap design did not notably affect the clinical parameters measured. Conversely, Castagna et al. [40] observed significant variations in CAL between their pre-operative assessments and follow-up periods. Specifically, while there were no substantial differences among flap designs regarding VPI, PPD at the first and second molar sites, and CAL at the second molar site, Castagna et al. [40] highlighted notable changes in CAL, particularly at the buccal and buccal–distal sites. These findings suggest that while flap design may not influence most periodontal outcomes, it could have a more pronounced effect on CAL over time, indicating a potential area for further investigation to refine surgical techniques and improve patient outcomes in lower third molar extractions.

It should also be noted, as a limitation of our study, that the average duration of piezoelectric surgery was generally slightly longer than that of conventional techniques using a drill handpiece because piezoelectric devices have a more limited cutting efficiency. It is important to note that conventional drills are faster and more efficient at cutting bone, resulting in faster procedures, while piezoelectric surgery offers greater precision and protection against damage to surrounding tissues; its slower cutting speed extends the overall operation time.

This study represents the first attempt to use an objective and reproducible method for collecting patients’ clinical parameters such as a craniometric distance evaluation. Consequently, this study contributes to the ongoing effort to obtain progressively more reliable data. Additionally, while craniometric distance acquisition is straightforward, the processing required to evaluate swelling requires a skilled operator and may be subject to a steep learning curve. In addition, all surgical procedures were performed by only a single trained oral surgeon (S.C.), so the results did not vary due to different operators’ skills. Considering the atraumatic nature of the piezosurgery technique and the absence of clinical complications, future research could consider further studies with large cohorts, greater clinical variability, and comprehensive analyses across groups and clinical aspects related to pre- and post-surgery, which would be necessary to enhance the reliability of the findings.

## 5. Conclusions

In conclusion, the comparative analysis of these two treatments revealed significant improvements in pain duration at sites treated with piezoelectric surgery compared to the conventional method. Longer surgical procedures generally result in more pain, which tends to increase with the complexity of the surgery. This is particularly true for the slower micrometric cutting action of the piezoelectric device. Additionally, pain following third molar extraction is influenced by several factors, including smoking, oral hygiene, age, anxiety, and a history of pericoronitis. Additionally, the swelling was consistently greater in all conventionally treated sites, with all measurements larger than those of the experimentally treated sites, as evidenced by a negative delta. Further studies from other centers are needed to substantiate these results.

## Figures and Tables

**Table 1:** Descriptive analysis of study population.

Variable	Study Population (N = 41)
Sex	
F	24 (58.5%)
M	17 (41.5%)
Age	10–61 (mean 29.2 y.o.)
Median (IQR)	24.0 (18.0, 38.0)
Type	
CL II B	14 (34.1%)
CL III C	13 (31.7%)
GERM	14 (34.1%)

**Table 2:** Linear mixed-model results are reported as means and 95 % confidence intervals (95%CI), with the relative *p*-values of models with time as the random effect. *p*-values from the linear mixed-effect model of time passing reveal a significant reduction for ΔAJME, ΔAJANS, ΔAJLLV, and ΔAJT from the 3rd to 7th day.

Variable	Mean (95 % CI)		
	3rd Day	7th Day	*p*-Value
Δ_AJME_	−1.390 (−1.575, −1.206)	−0.293 (−0.477, −0.108)	**<0.001**
Δ_AJANS_	−0.829 (−1.085, −0.5732)	−0.195 (−0.451, 0.061)	**<0.001**
Δ_AJLLV_	−0.805 (−0.997, −0.613)	−0.415 (−0.607, −0.223)	**<0.001**
0Δ_AJSLV_	−1.146 (−1.36, 0.935)	−0.951 (−1.16, −0.740)	0.160
Δ_AJNW_	−0.683 (−0.883, −0.483)	−0.512 (−0.712, −0.312)	0.233
Δ_AJT_	−0.951 (−1.108, −0.794)	−0.707 (−0.864, −0.550)	**0.031**

## Data Availability

The data used to support the findings of this study will be made available on request from the corresponding author.
